# Supplementary material for: De Novo Assembly-Based Analysis of RPGR Exon ORF15 in an Indigenous African Cohort Overcomes Limitations of a Standard Next-Generation Sequencing (NGS) Data Analysis Pipeline
Source: Genes (Basel). 2020 Jul 15;11(7):800. doi: 10.3390/genes11070800 (PMC7396994; doi:10.3390/genes11070800)
Supplement: Supplementary file 1 [file genes-11-00800-s001.zip › Figure_S1.docx]

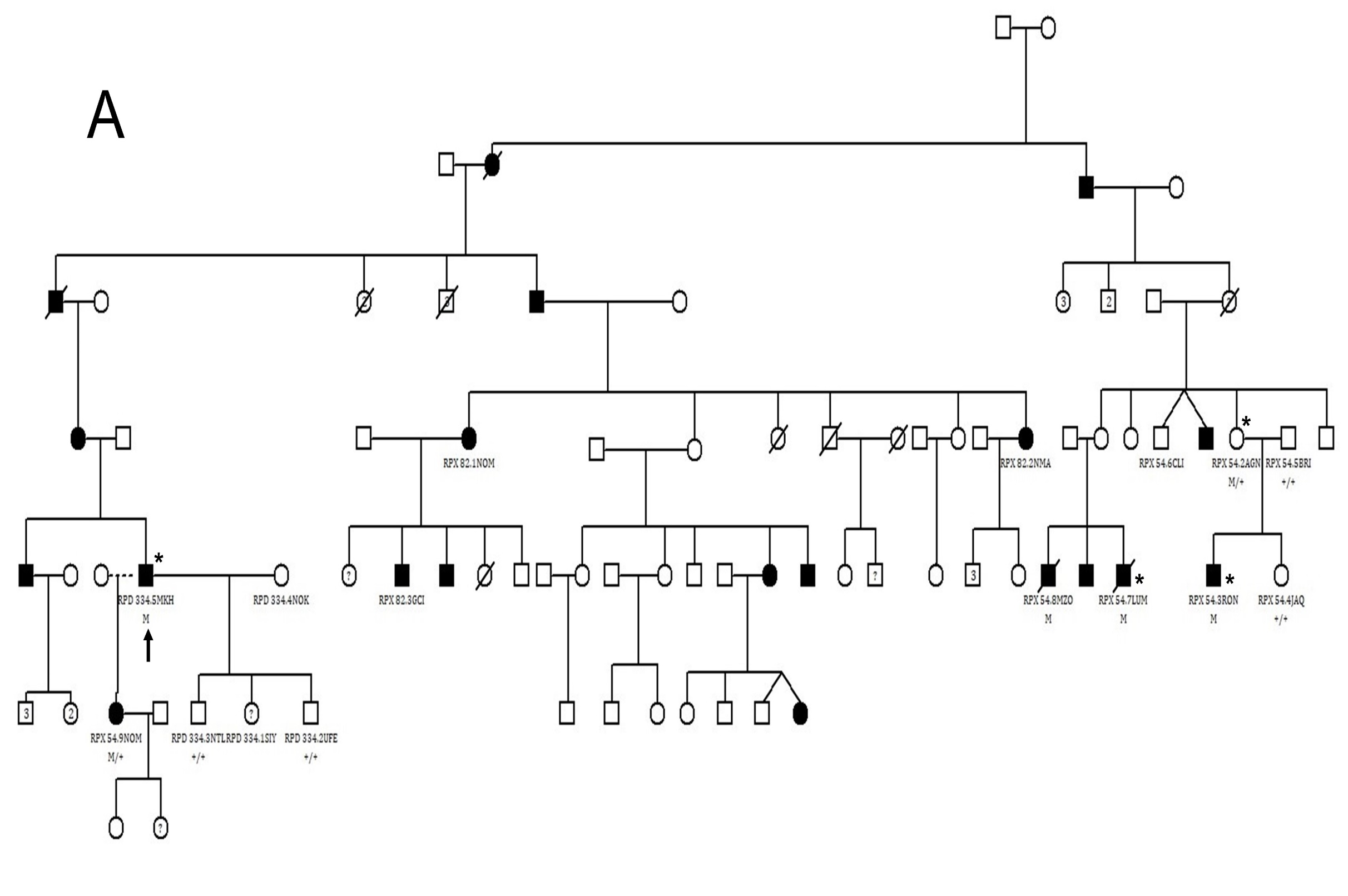


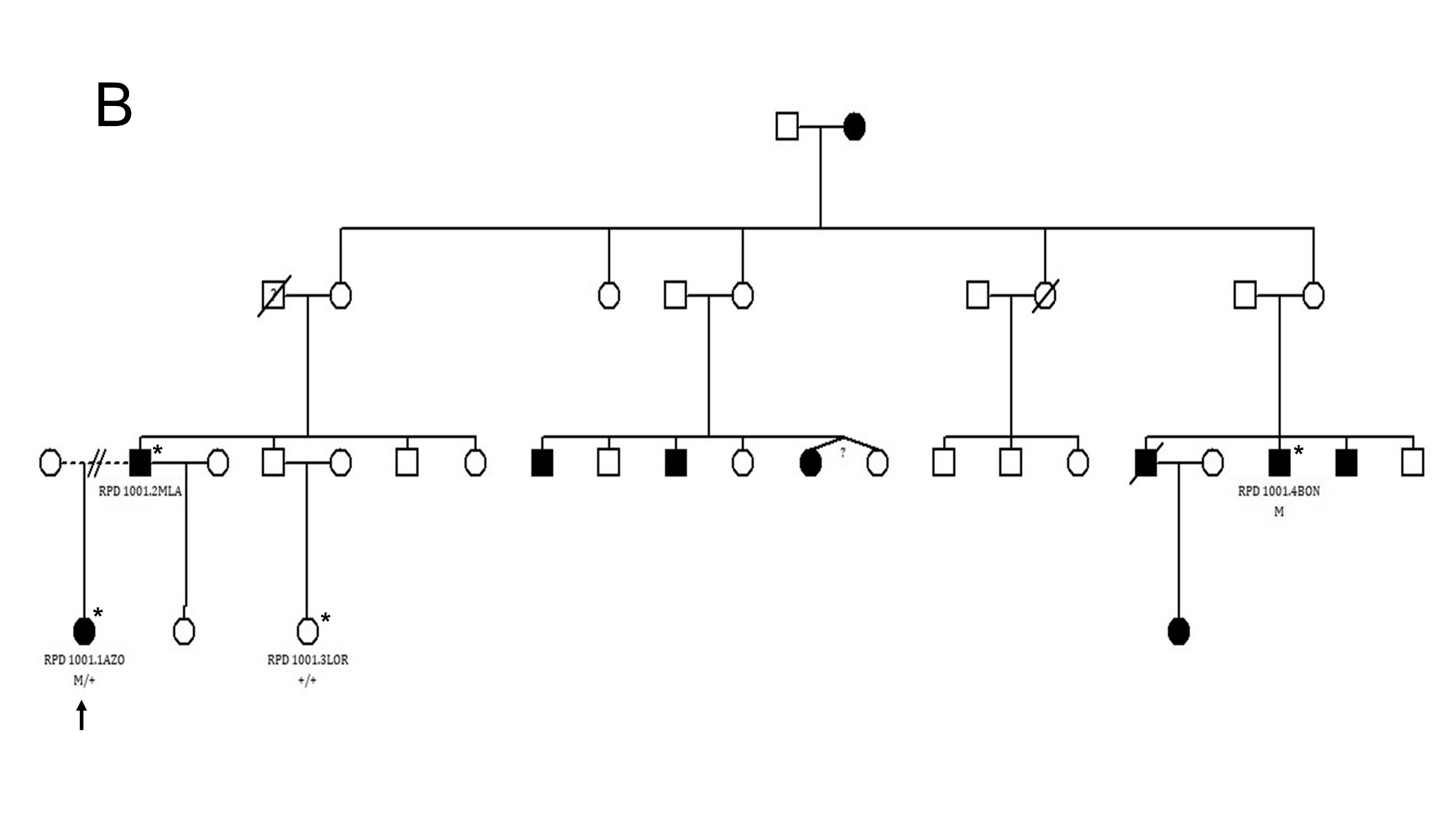


**
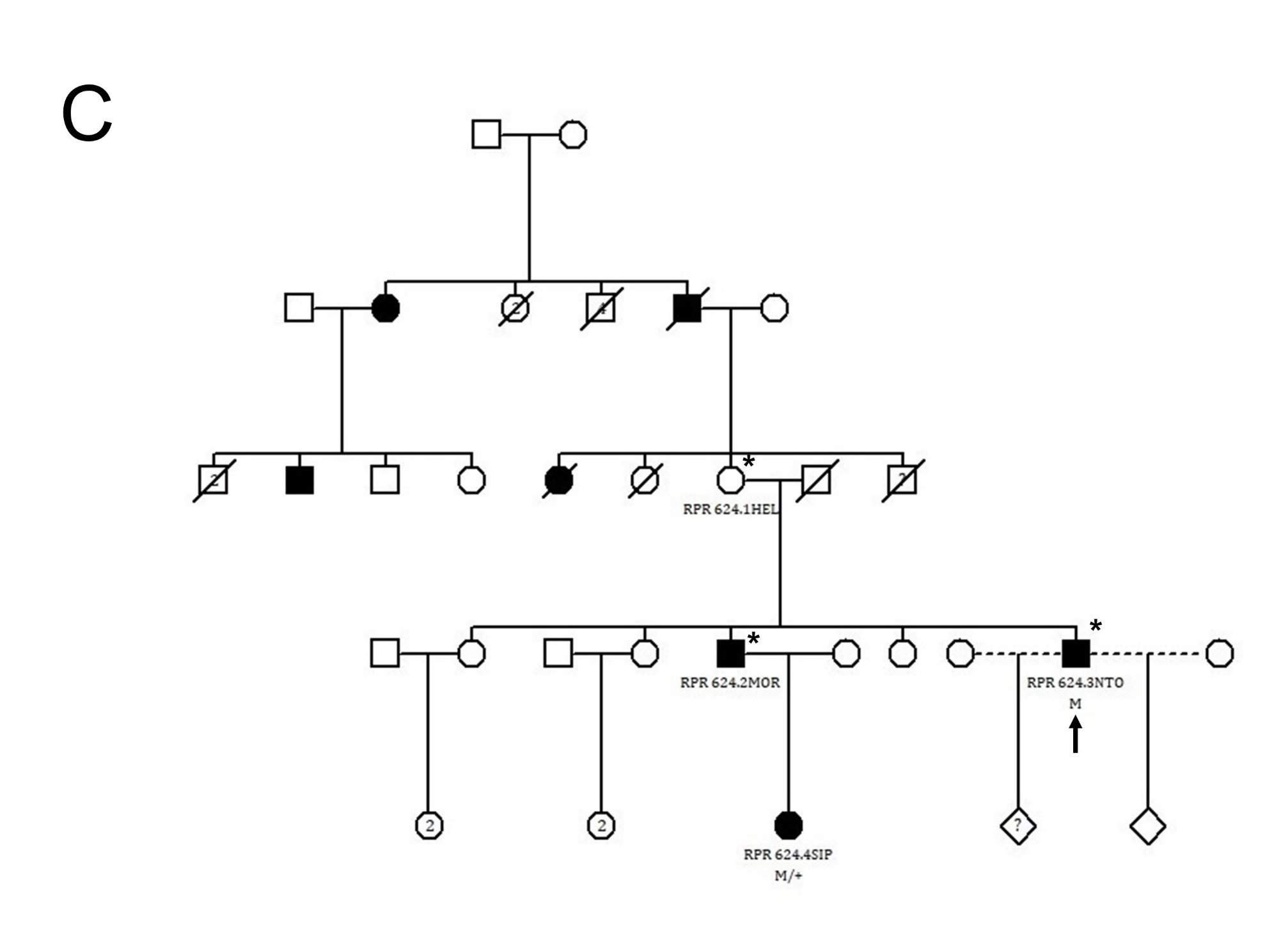
**

**Figure S1: Pedigrees of three families identified with *RPGR* ORF15 mutations, after WES failed to resolve the causal mutations*.*** Squares represent males, and circles, females. Shaded symbols indicate individuals affected with IRD, and a question mark highlights subjects for whom the clinical status is unknown. Identifier codes show individuals from whom biological material was available in the IRD registry, and an asterisk indicates samples subjected to WES. The individuals indicated with an arrow were selected for *RPGR* ORF15 sequencing; these persons in families A (RPD 54) and B (RPD 1001) were each found to carry the c.2790_2791delGG mutation, whilst the individual in family C (RPR 624) carried the c.2964_2965delGG mutation. These findings prompted the current study. Segregation of the mutation in the families is indicated as: M (hemizygous mutation); M/+ (heterozygous mutation); and +/+ (Wild Type).
