# Supplementary material for: De Novo Assembly-Based Analysis of RPGR Exon ORF15 in an Indigenous African Cohort Overcomes Limitations of a Standard Next-Generation Sequencing (NGS) Data Analysis Pipeline
Source: Genes (Basel). 2020 Jul 15;11(7):800. doi: 10.3390/genes11070800 (PMC7396994; doi:10.3390/genes11070800)
Supplement: Supplementary file 1 [file genes-11-00800-s001.zip › HTML_S3_RPGR_ORF15_analysis_pipeline.html]

RPGR\_ORF15\_analysis\_pipeline


# *RPGR* ORF15 long-range PCR data analysis pipeline¶

**Jordi Maggi¹**

***¹Institute of Medical Molecular Genetics, University of Zurich, 8952 Schlieren, Switzerland***

This JupyterLab notebook describes and collects all step of the data analysis needed for secondary data analysis of *RPGR*'s ORF15 sequences generated by the protocol described in the following publication:

**Li, J., Tang, J., Feng, Y., Xu, M., Chen, R., Zou, X., … Wong, L. J. C. (2016). Improved Diagnosis of Inherited Retinal Dystrophies by High-Fidelity PCR of ORF15 followed by Next-Generation Sequencing. Journal of Molecular Diagnostics, 18(6), 817–824. https://doi.org/10.1016/j.jmoldx.2016.06.007**

It is reccomended to install the necessary packages through the Anaconda distribution platform (https://www.anaconda.com). The packages and installation instructions can be found on BioConda (https://bioconda.github.io) or on the corresponding websites.

The required packages are:

- **JupyterLab** (https://jupyterlab.readthedocs.io/en/stable/#)
- **Python 3** (https://www.python.org)
- **SPAdes** (https://github.com/ablab/spades)
- **MAFFT v7** (https://mafft.cbrc.jp/alignment/software/)
- **SNP-sites** (https://www.sanger.ac.uk/science/tools/snp-sites)

In order for it to work, the folder where this JupyterLab notebook file is located needs to contain the following subfolders:

- **FASTQ** (containing the FASTQ files to be analyzed)
- **SPAdes** (where the assembly output files will be located)
  - **kmer\_99\_127** (subfolder where the first assembly step's output will be stored)
  - **selected** (subfolder where the contigs selected from the first step will be stored)
  - **trusted\_contig** (subfolder where the second and (possibly) third assemlby steps' output will be sotred)
  - **final\_contig** (subfolder where the final contig used in the next steps will be stored)
- **multi\_fasta** (where the fasta files used for alignment will be stored)
- **Aligned\_fasta** (where the aligned fasta and clustal files will be stored)
- **Annotation** (where files needed for annotation of the variants will be stored)
- **VCFs** (where the resulting variant files will be stored)

To run this pipeline, the user only need to make sure that the script is located in the correct folder with the correct folder structure (just specified), that the FASTQ files are named correctly (see below), that the **Annotation** folder contains the provided gnomAD file, and that the **samples** list specified in STEP 1 contains the correct sample IDs to be analyzed.

**Note**: .csv files created here are separated by semicolons **";"** and this is used to go through the data.

\*\*STEP 1: de novo assembly\*\*

The first step in this pipeline is the generation of contigs from the raw sequencing results (FASTQ files) with the de novo assembly software SPAdes (https://github.com/ablab/spades).

This step require FASTQ files as input. Here we specify all the FASTQ files to be analyzed. Make sure that all FASTQ are collected into one folder named FASTQ. Also, the FASTQ files should be named with this scheme:

**sampleID\_typeofdata\_numberofdataset-datasetkind.fastq**

The **sampleID** should not contain **"\_"** and should be unique. Also, it should be the same as the one provided in the **samples** list (in the next cell).
The **typeofdata** is specified in the SPADes manual (see below), briefly:

```
* pe for paired-end libraries
* mp for mate-pair libraries
* pacbio for PacBio CLR libraries
* pacbio-s for PacBio CCS libraries
```

The **numberofdataset** specifies how the datasets are organized:

```
* incremental integers as the first number (1,2,3,4, etc) for the order of datasets
```

The **datasetkind** specifies what kind of dataset it is:

```
* 12 for interlaced paired-end libraries (containing both right and left reads)
* 1 for right-only reads libraries
* 2 for left-only reads libraries
* m for merged reads from paired-end library
```

In [ ]:

```
#Make sure you have all the required FASTQ files saved in a subdirectory of the current working directory named "FASTQ"
files = !ls -R FASTQ
#To make sure that all the intended file names are stored in the "files" list, this command will print the list
print(files)

#Now specify the name of each sample to be run; 
#Either fill in the samples list manually and comment out the for loop lines, or
#Make sure that the FASTQ folder only contains files that need to be analyzed and use the for loop to create the samples list (just by running the cell as is)
samples = []

for file in files:
    sample = file.split('_')[0]
    if sample == "Icon":
        continue
    if sample not in samples:
        samples.append(sample)
    
print(samples, len(samples))
```

Our tests, showed that different datasets responded best to different k-mer settings. Generally, the best results were obtained with longer kmers. Therefore, the first assembly step will use kmer 99 and 127.
The output of this step will be a fasta file located in the **SPAdes/kmer\_99\_127** folder containing the resulting contigs for each sample named:

**sampleID\_contigs.fasta**

SPAdes' manual can be found here: http://cab.spbu.ru/files/release3.12.0/manual.html.

In [ ]:

```
for sample in samples:
    file_list = []
    for file in files:
        if sample == file.split('_')[0]:
            file_list.append('FASTQ/'+file)
    inputs = ''
    for file in file_list:
        dataset = file.split('_')
        inputs += '--' + dataset[1] + dataset[2].split('.')[0] + ' '
        inputs += file + ' '

    !spades.py {inputs}-k 99,127 --careful --cov-cutoff 20.0 -o SPAdes/kmer_99_127
    
    #SPAdes saves its output files as 'contigs.fasta', we therefore need to rename them including the sample ID
    !mv SPAdes/kmer_99_127/contigs.fasta SPAdes/kmer_99_127/{sample}_contigs.fasta
```

\*\*STEP 2: contigs selection and refinement\*\*

Here, we will use Python code to filter out the most promising contigs generated by the assembler for each sample and collect these into a new file named **sampleID\_selected\_contigs.fasta**. If the contig satisfies the G-content criterium and the length roughly corresponds to the expected amplicon size (2100 bp, >2000 or <2300 bp), this will be saved as the final\_contig for further analysis and the sample will not undergo further assembly steps.
On the other hand, if no satisfactory contig is found, then the sample ID will be added to the **sample\_incomplete** list that will undergo a second assembly step with different kmer settings.

In [ ]:

```
def ReverseComplement1(seq):
    seq_dict = {'A':'T','T':'A','G':'C','C':'G'}
    return "".join([seq_dict[base] for base in reversed(seq)])

k99_127_contigs = !ls -R SPAdes/kmer_99_127

samples_incomplete = []

for sample in samples:
    sample_files = []
    for file in k99_127_contigs:
        if sample == file.split('_')[0] and file.split('_')[1] == 'contigs.fasta':
            fyle = open('SPAdes/kmer_99_127/{}'.format(file), 'r')
            content = fyle.readlines()
            sample_files.append(content)

    kmers = ['99/127']
    
    #Create the selected_contigs and final_contigs files for the sample
    selected_contigs_file = open('SPAdes/selected/{}_selected_contigs.fasta'.format(sample), 'w')
    final_contigs_file = open('SPAdes/final_contig/{}_final_contigs.fasta'.format(sample), 'w')
    
    for i in range(len(sample_files)):
        contigs_score = []
        for i2 in range(len(sample_files[i])):
            if sample_files[i][i2][0] == '>':
                contig = ''
                length = int(sample_files[i][i2].split('_')[3])
                coverage = float(sample_files[i][i2].split('_')[5])
                if length < 400: #Minimum length of 400 to be considered
                    continue
                if length > 2300: #Not considering contigs that are too large
                    continue
                if coverage < 30: #Minimum coverage of 30 to be considered
                    continue
                for i3 in range(i2+1,len(sample_files[i])):
                    if sample_files[i][i3][0] == '>':
                        break
                    else:
                        contig += sample_files[i][i3].strip()
                #The reference seq on the correct strand has 43% G content, the reverse has 7%.
                #Since MAFFT cannot reverse complement, it's best to do it here when needed. 
                #When G_content < 15% seems like a good threshold
                G_count = contig.count('G')
                G_content = G_count/len(contig)
                if G_content > 0.15:
                    seq1 = ReverseComplement1(contig)
                else:
                    seq1 = contig
                new_G_count = seq1.count('G')
                new_G_content = new_G_count/len(seq1)
                if new_G_content > 0.15:
                    continue
                score = length*2 + coverage
                contigs_score.append((score, sample_files[i][i2], seq1))        
        contigs_score.sort(reverse=True)
        if len(contigs_score) > 5:
            for score in contigs_score[:6]: #Considering only the contigs with the best 5 scores
                if len(score[2]) > 2040 and len(score[2]) < 2300:
                    final_contigs_file.write('{}_kmer_{}\n'.format(score[1].strip(), kmers))
                    final_contigs_file.write('{}'.format(score[2]))
                    final_contigs_file.write('\n')
                    ref = score[2]
                    print('Final contig for {}: length {}, coverage {}'.format(sample, len(score[2]), int(score[0]-2*len(score[2]))))
                    break
                else:
                    selected_contigs_file.write('{}_kmer_{}\n'.format(score[1].strip(), kmers[i]))
                    selected_contigs_file.write('{}'.format(score[2]))
                    selected_contigs_file.write('\n')
                if sample not in samples_incomplete:
                    samples_incomplete.append(sample)
                
        else:
            for score in contigs_score: #Considering all the contigs when there are less than 6
                if len(score[2]) > 2040 and len(score[2]) < 2300:
                    final_contigs_file.write('{}_kmer_{}\n'.format(score[1].strip(), kmers))
                    final_contigs_file.write('{}'.format(score[2]))
                    final_contigs_file.write('\n')
                    ref = score[2]
                    print('Final contig for {}: length {}, coverage {}'.format(sample, len(score[2]), int(score[0]-2*len(score[2]))))
                    break
                else:
                    selected_contigs_file.write('{}_kmer_{}\n'.format(score[1].strip(), kmers[i]))
                    selected_contigs_file.write('{}'.format(score[2]))
                    selected_contigs_file.write('\n')
                if sample not in samples_incomplete:
                    samples_incomplete.append(sample)
    
    selected_contigs_file.close()
    final_contigs_file.close()
print(samples_incomplete)
```

\*\*STEP 3: repeating assembly using different kmers and the RPGR ORF15 reference sequence and the previously selected contigs as trusted contigs\*\*

The assembly is run again for the incomplete samples by using different kmer settings (kmers 33, 55, 77, 99) and the selected contig and RPGR reference sequence as trusted contigs in SPAdes.

In [ ]:

```
samples_incomplete_2 = []

for sample in samples_incomplete:
    contigs_file = 'SPAdes/selected/'+sample+'_selected_contigs.fasta'
    RPGR_ref_contig = 'SPAdes/RPGR_orf15_LR_PCR_sequence.fasta'
    file_list = []
    for file in files:
        if sample == file.split('_')[0]:
            file_list.append('FASTQ/'+file)
    inputs = ''
    for file in file_list:
        dataset = file.split('_')
        inputs += '--' + dataset[1] + dataset[2].split('.')[0] + ' '
        inputs += file + ' '
    !spades.py {inputs}-k 33,55,77,99 --careful --cov-cutoff 20.0 --trusted-contigs {RPGR_ref_contig} --trusted-contigs {contigs_file} -o SPAdes/trusted_contig
    !mv SPAdes/trusted_contig/contigs.fasta SPAdes/trusted_contig/{sample}_trusted_kmer33_55_77_99_contigs.fasta
    try:
        file = open('SPAdes/trusted_contig/{}_trusted_kmer33_55_77_99_contigs.fasta'.format(sample))
        file.close()
    except:
        samples_incomplete_2.append(sample)
```

\*\*STEP 4: kmer 99 final contig selection\*\*

In this step, the final contig is selected from the latest SPAdes run files. If no satisfactory contig could be identified, the **sampleID** is added to the **samples\_incomplete\_2** list for a third and final assembly step.

In [ ]:

```
def ReverseComplement1(seq):
    seq_dict = {'A':'T','T':'A','G':'C','C':'G'}
    return "".join([seq_dict[base] for base in reversed(seq)])

trusted_contigs = !ls -R SPAdes/trusted_contig


for sample in samples_incomplete:
    sample_files = []
    for file in trusted_contigs:
        if sample == file.split('_')[0] and file.split('_')[1] == 'trusted' and file.split('_')[2] == 'kmer33':
            fyle = open('SPAdes/trusted_contig/{}'.format(file), 'r')
            content = fyle.readlines()
            sample_files.append(content)

    kmers = ['33/55/77/99']
    
    #Create the selected_contigs file for the sample
    final_contigs_file = open('SPAdes/final_contig/{}_final_contigs.fasta'.format(sample), 'w')
    
    for i in range(len(sample_files)):
        contigs_score = []
        for i2 in range(len(sample_files[i])):
            if sample_files[i][i2][0] == '>':
                contig = ''
                length = int(sample_files[i][i2].split('_')[3])
                coverage = float(sample_files[i][i2].split('_')[5])
                if length < 1500: #Minimum length of 1500 to be considered
                    continue
                if length > 2300: #Not considering contigs that are too large
                    continue
                if coverage < 30: #Minimum coverage of 30 to be considered
                    continue
                for i3 in range(i2+1,len(sample_files[i])):
                    if sample_files[i][i3][0] == '>':
                        break
                    else:
                        contig += sample_files[i][i3].strip()
                #The reference seq on the correct strand has 43% G content, the reverse has 7%.
                #Since MAFFT cannot reverse complement, it's best to do it here when needed. 
                #When G_content < 15% seems like a good threshold
                G_count = contig.count('G')
                G_content = G_count/len(contig)
                if G_content > 0.15:
                    seq1 = ReverseComplement1(contig)
                else:
                    seq1 = contig
                new_G_count = seq1.count('G')
                new_G_content = new_G_count/len(seq1)
                if new_G_content > 0.15:
                    continue
                score = length*2 + coverage
                contigs_score.append((score, sample_files[i][i2], seq1))
                
        contigs_score.sort(reverse=True)
        if len(contigs_score) > 5:
            for score in contigs_score[:6]: #Considering only the contigs with the best 5 scores
                if len(score[2]) > 2040 and len(score[2]) < 2300:
                    final_contigs_file.write('{}_kmer_{}\n'.format(score[1].strip(), kmers))
                    final_contigs_file.write('{}'.format(score[2]))
                    final_contigs_file.write('\n')
                    print('Final contig for {}: length {}, coverage {}'.format(sample, len(score[2]), int(score[0]-2*len(score[2]))))
                    break
                else:
                    final_contigs_file.write('{}_kmer_{}_incomplete\n'.format(score[1].strip(), kmers))
                    final_contigs_file.write('{}'.format(score[2]))
                    final_contigs_file.write('\n')
                    print('It was not possible to contruct a contig to cover the entire amplicon for sample {}'.format(sample))
                    samples_incomplete_2.append(sample)
                    
        else:
            for score in contigs_score: #Considering all the contigs when there are less than 6
                if len(score[2]) > 2040 and len(score[2]) < 2300:
                    final_contigs_file.write('{}_kmer_{}\n'.format(score[1].strip(), kmers))
                    final_contigs_file.write('{}'.format(score[2]))
                    final_contigs_file.write('\n')
                    print('Final contig for {}: length {}, coverage {}'.format(sample, len(score[2]), int(score[0]-2*len(score[2]))))
                    break
                else:
                    final_contigs_file.write('{}_kmer_{}_incomplete\n'.format(score[1].strip(), kmers))
                    final_contigs_file.write('{}'.format(score[2]))
                    final_contigs_file.write('\n')
                    print('It was not possible to contruct a contig to cover the entire amplicon for sample {}'.format(sample))
                    samples_incomplete_2.append(sample)
    
    final_contigs_file.close()
```

\*\*STEP 5: using different kmer settings for samples with no complete contig\*\*

We now repeat once more a SPAdes run for the samples that could not be solved with the previous assembly settings. In this case, we use kmer 77, 99, and 127 and the previously generated contigs and RPGR reference sequence as input contigs.

In [ ]:

```
print(samples_incomplete_2)
for sample in samples_incomplete_2:
    try:
        fyle = open('SPAdes/final_contig/{}_final_contigs.fasta'.format(sample))
        contigs_file = 'SPAdes/final_contig/'+sample+'_final_contigs.fasta'
        fyle.close()
    except:
        contigs_file = 'SPAdes/selected/'+sample+'_selected_contigs.fasta'
    RPGR_ref_contig = 'SPAdes/RPGR_orf15_LR_PCR_sequence.fasta'
    file_list = []
    for file in files:
        if sample == file.split('_')[0]:
            file_list.append('FASTQ/'+file)
    inputs = ''
    for file in file_list:
        dataset = file.split('_')
        inputs += '--' + dataset[1] + dataset[2].split('.')[0] + ' '
        inputs += file + ' '
    !spades.py {inputs}-k 77,99,127 --careful --cov-cutoff 20.0 --untrusted-contigs {contigs_file} --trusted-contigs {RPGR_ref_contig} -o SPAdes/trusted_contig
    !mv SPAdes/trusted_contig/contigs.fasta SPAdes/trusted_contig/{sample}_trusted_kmer77_99_127_contigs.fasta
```

\*\*STEP 6: final contig selection 2\*\*

In this step, the final contig is selected from the latest SPAdes run file.

In [ ]:

```
def ReverseComplement1(seq):
    seq_dict = {'A':'T','T':'A','G':'C','C':'G'}
    return "".join([seq_dict[base] for base in reversed(seq)])

trusted_contigs = !ls -R SPAdes/trusted_contig


for sample in samples_incomplete_2:
    sample_files = []
    for file in trusted_contigs:
        if sample == file.split('_')[0] and file.split('_')[1] == 'trusted' and file.split('_')[2] == 'kmer77':
            fyle = open('SPAdes/trusted_contig/{}'.format(file), 'r')
            content = fyle.readlines()
            sample_files.append(content)

    kmers = ['77/99/127']
    
    #Create the selected_contigs file for the sample
    final_contigs_file = open('SPAdes/final_contig/{}_final_contigs.fasta'.format(sample), 'w')
    
    for i in range(len(sample_files)):
        contigs_score = []
        for i2 in range(len(sample_files[i])):
            if sample_files[i][i2][0] == '>':
                contig = ''
                length = int(sample_files[i][i2].split('_')[3])
                coverage = float(sample_files[i][i2].split('_')[5])
                if length < 1500: #Minimum length of 1500 to be considered
                    continue
                if length > 2300: #Not considering contigs that are too large
                    continue
                if coverage < 30: #Minimum coverage of 30 to be considered
                    continue
                for i3 in range(i2+1,len(sample_files[i])):
                    if sample_files[i][i3][0] == '>':
                        break
                    else:
                        contig += sample_files[i][i3].strip()
                #The reference seq on the correct strand has 43% G content, the reverse has 7%.
                #Since MAFFT cannot reverse complement, it's best to do it here when needed. 
                #When G_content < 15% seems like a good threshold
                G_count = contig.count('G')
                G_content = G_count/len(contig)
                if G_content > 0.15:
                    seq1 = ReverseComplement1(contig)
                else:
                    seq1 = contig
                new_G_count = seq1.count('G')
                new_G_content = new_G_count/len(seq1)
                if new_G_content > 0.15:
                    continue
                score = length*2 + coverage #I give double importance to the length compared to the coverage
                contigs_score.append((score, sample_files[i][i2], seq1))
                
        contigs_score.sort(reverse=True)
        if len(contigs_score) > 5:
            for score in contigs_score[:6]: #Considering only the contigs with the best 5 scores
                if len(score[2]) > 2040 and len(score[2]) < 2300:
                    final_contigs_file.write('{}_kmer_{}\n'.format(score[1].strip(), kmers))
                    final_contigs_file.write('{}'.format(score[2]))
                    final_contigs_file.write('\n')
                    ref = score[2]
                    print('Final contig for {}: length {}, coverage {}'.format(sample, len(score[2]), score[0]-2*len(score[2])))
                    break
                else:
                    final_contigs_file.write('{}_kmer_{}_incomplete\n'.format(score[1].strip(), kmers))
                    final_contigs_file.write('{}'.format(score[2]))
                    final_contigs_file.write('\n')
                    print('It was not possible to contruct a contig to cover the entire amplicon for sample {}'.format(sample))

                    
        else:
            for score in contigs_score: #Considering all the contigs when there are less than 6
                if len(score[2]) > 2040 and len(score[2]) < 2300:
                    final_contigs_file.write('{}_kmer_{}\n'.format(score[1].strip(), kmers))
                    final_contigs_file.write('{}'.format(score[2]))
                    final_contigs_file.write('\n')
                    ref = score[2]
                    print('Final contig for {}: length {}, coverage {}'.format(sample, len(score[2]), int(score[0]-2*len(score[2]))))
                    break
                else:
                    final_contigs_file.write('{}_kmer_{}_incomplete\n'.format(score[1].strip(), kmers))
                    final_contigs_file.write('{}'.format(score[2]))
                    final_contigs_file.write('\n')
                    print('It was not possible to contruct a contig to cover the entire amplicon for sample {}'.format(sample))

    
    final_contigs_file.close()
```

\*\*STEP 7: creating alignment input files\*\*

Now we need to create a new multi-FASTA file containing the reference *RPGR* ORF15 sequence and the selected contigs from the current sample, which is needed by the aligner (MAFFT v7, see **STEP 4**)

In [ ]:

```
RPGR_ORF15_PCR_sequence = 'TGTCTTTGGCTCCTTAACACAGCTGCATCAGTTGCttttttttttACTACACATAAAATAATTGACATAAAATCAATTTAATAACACGTAATGAGTGCCCGTTATATGCAAGGCATTTAAATTGTCTGACTGGCCATAATCGGGTCACATTTAAGGTTTGTTACTTCAATTCCAAGTAATGTGGTAATACATTATTCCAGAACTTTTTGGAACCTGATGGCCCGTTTTTTAAAAGTCGTTTTGACTGGACTGGCATTTTGGACCTCTGCTCTTTCCCATTTCCCTGTGTGTTAGTAACTGACTTTTTTTGATATGTTTTATGTTTGCCATATTTCACAGATCCTTTTATTTTGCTCACTTTTTTGTACTCCTCTCCATCCTGCCTTTCATTCTCTTCTTCGCctgtctcctgatacttcccctcttcttcctcctcctcttctctgttcctcctgttttcttctccttccccctccttttccctttcttctccttcctcctctccttcctcttcctctccttccccctctccttcctccccttccacctccccttccacttccccttcctcttcttcctccccttctccctccccttcttcctctccctctccttcttcctccccttcttcttccccttcctcctcttccccctccccttctccttcctcctcttccccctccccttctccttcctccccttccccttctccttcctcttccccctccccttctccttcctcctcttccccctcccattctccttcctcctcttccccctccccttctccatcctccccttccccttctccttcctcctcttccccctccccttctccttcctctccttcctcctcccctttcccttctccttcctcctcttctccctccccttctccttcctcttctccctccccttctccttcctcctcttctccctccccttctccttcctcttctccctccccttctccttcctcctcttccccctccccttctccttcctccccttcttcctccccttctccttcttccccttcttcctcccctttcccttctccttcctcctcttccccctccccttcctcctcttccccctccccttcctcctcttccccctcaccctcctcctcttcctcttccctctctcctttcccctcctctacttcccctccctctacttcccctccctcctctttttcctcccctctcccctctgtttcctcctcttccccctctccttggtctccttcttcctctcctttctcctccttccccgctctttcctcctttttcctctctccttcctccttttcacgttctccctccacttcttccccttctccttcctctttcccttctccctccttctcttcttcctcttctctgtctccctcctcttcttctccttctccatgctcctcctcccctccctcctccatctcttggtttctttcctTCTGATGGCCCTGCTCCCTCTCCTTTTGCTCCTGctcttccccatccctcttcttccattcttccttctctgctagttccttctctccctctcctGGCCTCTCCATTTCTCCTCTACCCTTGTCTTTCTCCCCCTTCTCCCTCTCCTCATCTTGCCAGTGTTCTGCTCCTGAACTACCTTCCTCACAGGTTCCATCCCCTCTACCTTCAGGCCCATCCTCTGCTTCTCCCACTGATTTTGCCTTGCCTTCACTCACCTCTGCTTTGTCTGTAAGGTCATCTGATAGGATCTCTGTTTTCTCCTTTCTTCCTCCATGCACCTTCACATTTTCCTCATTTGCTTCTACCTCTTGCTCCTCTATTCCATTTCCTTTTGAATCCTCTGCTCCTTCCTTCTCCTCTGGGATCTCTGACAAGCGATCACATTTAAAATCATACTTTGCCATGGATTTGGATATTATGGGTTTAGTCTCTTTTTCATTTCCTTGTTCAATCAATTTCCTGCCATACCGTATGTTTTGGTCAGTTTCCACTTCATCATCCTCTTTCTCTCCACTACTAAATTCTATTGCCTCAGGCTGCTGGAATCCATCAGCTATACCCTGTTGACTCTCACTTGCACCTTCCATGTAACTGTCTGGCT'
reference_header = '>RPGR_ORF15_lrPCR_2.1kb_reference_sequence'

for sample in samples:
    #Create the new multi-FASTA file
    multi_fasta = open('multi_fasta/{}_contigs_RPGR.fasta'.format(sample), 'w')
    contigs_file = open('SPAdes/final_contig/{}_final_contigs.fasta'.format(sample), 'r')
    contigs = contigs_file.readlines()
    contigs_file.close()
    #Writing the RPGR reference sequence first
    multi_fasta.write('{}\n'.format(reference_header))
    multi_fasta.write('{}\n'.format(RPGR_ORF15_PCR_sequence))
    
    #Now writing the contigs contained in the contigs_file to the multi_fasta file
    for contig in contigs:
        multi_fasta.write('{}'.format(contig))
        
    multi_fasta.close()
```

\*\*STEP 8: alignment\*\*

This step requires a multi-FASTA input file made up of at least two FASTA sequences to be aligned to one another. Running this will generate two different output files from one FASTA file, namely another FASTA file with the modified sequences with the alignment corrections. The second file is a CLUSTAL file (https://en.wikipedia.org/wiki/Clustal#Clustal\_and\_ClustalV), which shows the aligned sequences next to each other and highlights similarities and differences between the sequences. This step requires MAFFT v7 (https://mafft.cbrc.jp/alignment/software/).

The manual can be found here: https://mafft.cbrc.jp/alignment/software/manual/manual.html.

In [ ]:

```
for sample in samples:
    !mafft --auto multi_fasta/{sample}_contigs_RPGR.fasta > Aligned_fasta/{sample}_aligned.fasta
    !mafft --auto --clustalout multi_fasta/{sample}_contigs_RPGR.fasta > Aligned_fasta/{sample}_aligned.clustal
```

\*\*STEP 9: SNV calling\*\*

Next, single nucleotide variants present in the sample will be called from the aligned multi-FASTA file generated by MAFFT v7. SNV calling is done with SNP-sites (https://www.sanger.ac.uk/science/tools/snp-sites); as the name suggests, this algorithm only identifies SNVs (but no indels).

In [ ]:

```
for sample in samples:
    !snp-sites -v -o VCFs/{sample}_snp-sites_output.vcf Aligned_fasta/{sample}_aligned.fasta
```

\*\*STEP 10: Indels calling, variants collection and hg19 coordinates conversion\*\*

Because of its low-complexity, *RPGR*'s ORF15 is particularly prone to indels. It is therefore of capital importance to analyse this variant type, too.
Since I was not able to find an out-of-the-box tool to do so, a Python script will be used to do that.
This step also converts the local *RPGR* amplicon coordinates to the hg19 genomic build coordinates (**gNomen**). To do so, the aligned fasta file will be used. The first nucleotide (skipping **'-'** that precedes it) in the reference sequence will correspond to position 38144632 on chromosome X (hg19).

In [ ]:

```
hg19_correction = 38144632

hg19_sample_queries = {}

for sample in samples:
    SNV_lines = []
    try:
        fyle = open("VCFs/{}_snp-sites_output.vcf".format(sample), 'r')
        SNV_lines = fyle.readlines()
        fyle.close()
    except:
        print(sample, 'No SNVs found')

    alignment = open("Aligned_fasta/{}_aligned.fasta".format(sample), 'r')
    alignments = alignment.readlines()
    alignment.close()
    
    contigs_starts = []
    contig = ''
    contigs = []
    #Use the reference contig (ref) to get the correct position by substracting .count('-') to the aligned positions
    ref = ''
    
    for i in range(1,len(alignments)):
        if '>' in alignments[i]:
            break
        else:
            ref += alignments[i].upper().strip()
            
    #Creating the contigs
    for i in range(1,len(alignments)): #Skipping the first line because it containg the RPGR header
        if '>' not in alignments[i]:
            contig += alignments[i].strip()
        elif '>' in alignments[i]:
            contigs.append(contig.upper())
            contig = ''
        if i == len(alignments)-1:
            contigs.append(contig.upper())
            
    hg19_query_lines = []
    
    #Now screening contigs for dels and ins
    for i in range(len(contigs)):
        finish = 0
        start = 0
        contig_started = False
        for i3 in range(len(contigs[i])):
            if (finish - i3) > 0:
                continue
            elif contig_started == False:
                if contigs[i][i3] != '-':
                    contig_started = True
            elif contigs[i][i3] == '-' and contig_started == True and (i3-ref[:start].count('-')) < 2100:
                start = i3
                for i4 in range(i3,len(contigs[i])):
                    if contigs[i][i4] != '-':
                        finish = i4
                        break
                    else:
                        continue
                if i4 == len(contigs[i])-1:
                    continue
                hg19_start = start-ref[:start].count('-')+hg19_correction+1
                hg19_end = finish-ref[:start].count('-')+hg19_correction+1
                if i > 0:
                    if hg19_start != hg19_end and hg19_end - hg19_start > 1:
                        hg19_gNomen = 'chrX:g.'+str(hg19_start)+'_'+str(hg19_end-1)+'del'
                        hg19_query_lines.append((hg19_start,'X-'+str(hg19_start-1)+'-'+ref[i3-1:i4]+'-'+ref[i3-1], hg19_gNomen))
                    else:
                        hg19_gNomen = 'chrX:g.'+str(hg19_start)+'del'
                        hg19_query_lines.append((hg19_start,'X-'+str(hg19_start-1)+'-'+ref[i3-1:i4]+'-'+ref[i3-1], hg19_gNomen))

                elif i == 0:
                    length = hg19_end - hg19_start
                    #Checking if it is a duplication
                    for contig in contigs[1:]:
                        if '-' not in contig[start:finish]:
                            if contig[start:finish] == contig[finish:finish+length]:
                                hg19_gNomen = 'chrX:g.'+str(hg19_start)+'_'+str(hg19_end-1)+'dup'
                            else:
                                hg19_gNomen = 'chrX:g.'+str(hg19_start)+'_'+str(hg19_start+1)+'ins'+contig[start:finish]
                            hg19_query_lines.append((hg19_start,'X-'+str(hg19_start-1)+'-'+contig[i3-1]+'-'+contig[i3-1:i4], hg19_gNomen))

                                
                   
    #Now converting the SNVs data
    if len(SNV_lines) > 3:
        for line in SNV_lines[4:]:
            pos = int(line.split('\t')[1])
            hg19_start = pos-ref[:pos].count('-') + hg19_correction
            ref_n = line.split('\t')[3]
            alt = line.split('\t')[4]
            hg19_gNomen = 'chrX:g.'+str(hg19_start)+ref_n+'>'+alt
            hg19_query_lines.append((hg19_start, 'X-'+str(hg19_start)+'-'+ref_n+'-'+alt, hg19_gNomen))

    hg19_query_lines.sort()
    
    #Adding the variants info to the all_variants dictionary which will contain all samples and their variants
    #to be written onto a new file in the next step
    
    hg19_sample_queries[sample] = hg19_query_lines
```

\*\*STEP 11: VCF generation with gnomAD annotation (where available)\*\*

This step checks whether the variant is present in the gnomAD annotation file and, if so, it saves the **cNomen**, **pNomen**, and **frequency data**.
It then creates a VCF file containing the data (in .csv format) for each sample named **sampleID\_RPGR\_ORF15\_hg19\_variants.csv**.
Finally, if the variant is not present in the gnomAD file, it checks whether it is present in the **Unknown\_variants\_annotated.csv**, and if not, it writes it onto a file named **Unknown\_variants\_query\_lines.csv**.

In [ ]:

```
hg19_gnomad_file = open('Annotation/gnomAD_hg19.csv')
hg19_gnomad_lines = hg19_gnomad_file.readlines()
hg19_gnomad_file.close()

try:
    unknown_variants_file = open('Annotation/Unknown_variants_annotated.csv', 'r', encoding='utf-8')
    unknown_variants_annotated = unknown_variants_file.readlines()
    unknown_variants_file.close()
except:
    unknown_variants_annotated = []
    print('No annotation file for unknown variants present')

unknown_variants = {}

for sample in samples:
    hg19_file = open('VCFs/{}_RPGR_ORF15_hg19_variants.csv'.format(sample), 'w')
    hg19_variants_pos = []
    hg19_variants = {}
    hg19_file.write('chr;gNomen;cNomen;pNomen;gnomAD (%);rsID\n')
    Found = False
    for i in range(len(hg19_sample_queries[sample])):
        Found = False
        hg19_query_data = hg19_sample_queries[sample][i][1].split('-')
        hg19_gNomen = hg19_sample_queries[sample][i][2]
        hg19_pos = hg19_query_data[1]
        for hg19_gnomad in hg19_gnomad_lines[1:]:
            hg19_gnomad_data = hg19_gnomad.split(',')
            try:
                hg19_gnomad_data.remove('"gnomAD Exomes')
            except:
                "Item not present in the list"
            if int(hg19_pos) == int(hg19_gnomad_data[1]):
                if hg19_query_data[2] == hg19_gnomad_data[3] and hg19_query_data[3] == hg19_gnomad_data[4]:
                    Found = True
                    if hg19_pos == '38146601':
                        hg19_gnomad_data[10] = 'c.1754-103C>T'
                    if len(hg19_gnomad_data[9].split('(')) > 1:
                        pNomen = hg19_gnomad_data[9].split('(')[1][:-1]
                    else:
                        pNomen = hg19_gnomad_data[9]
                        if hg19_pos == '38146601':
                            pNomen = ''
                    hg19_file.write('X;{};NM_001034853.2:{};{};{:.4f};{}\n'.format(hg19_gNomen, hg19_gnomad_data[10], pNomen, float(hg19_gnomad_data[15])*100, hg19_gnomad_data[2]))
        if Found:
            continue
        else:
            not_found = True
            for line in unknown_variants_annotated[1:]:
                unknown_variant_info = line.strip().split(';')
                if hg19_sample_queries[sample][i][1] == unknown_variant_info[0]:
                    not_found = False
                    hg19_file.write('X;{};NM_001034853.2:{};{};NA;\n'.format(hg19_gNomen, unknown_variant_info[1], unknown_variant_info[2]))
            if not_found:        
                hg19_file.write('X;{};;;NA;\n'.format(hg19_gNomen))
                if hg19_sample_queries[sample][i][1] not in unknown_variants:
                    unknown_variants[hg19_sample_queries[sample][i][1]] = [sample]
                else:
                    unknown_variants[hg19_sample_queries[sample][i][1]].append(sample)

    hg19_file.close()
    
#Collect unknown variants in a file
new_variants = open('Unknown_variants_query_lines.csv', 'w')
for variant in unknown_variants:
    new_variants.write('{}'.format(variant))
    for sample in unknown_variants[variant]:
        new_variants.write(';{}'.format(sample))
    new_variants.write('\n')
new_variants.close()
```

The VCF file created will contain all variants in gNomen, but cNomen, pNomen and frequency data will be available only for variants present in the gnomAD file (or in the **Unknown\_variants\_annotated.csv** file).
For variants that are not present in gnomAD, the **Unknown\_variants\_query\_lines.csv** file contains the variants that need manual annotation. The query lines provided can be used, for example, on Varsome (https://varsome.com) to get the cNomen and pNomen (in the **Trascript** section), on top of other data.

If the analysis will be repeated, it is recommended to fill in the **Unknown\_variants\_annotated.csv** file found in the **Annotation** folder containing the annotation of gnomAD unknown variants identified in previous analysis so that manual annotation does not need to be repeated.
